# Mathematical Modeling of NaCl Scaling Development in Long-Distance Membrane Distillation for Improved Scaling Control

**DOI:** 10.3390/ma17153629

**Published:** 2024-07-23

**Authors:** Jingcheng Cai, Xingsen Mu, Jian Xue, Jiaming Chen, Zeman Liu, Fei Guo

**Affiliations:** 1 School of Energy and Power Engineering, Dalian University of Technology, Dalian 116024, China; wuyouwubu252@foxmail.com (J.C.); muxingsen@dlut.edu.cn (X.M.); cjm00000@mail.dlut.edu.cn (J.C.); coolee444@gmail.com (Z.L.); 2 Guangdong Provincial Key Laboratory of Green Chemical Product Technology, School of Chemistry and Chemical Engineering, South China University of Technology, Guangzhou 510640, China; xuejian@scut.edu.cn

**Keywords:** membrane distillation, membrane interface, long-distance process, NaCl scaling, scaling development

## Abstract

Membrane distillation is a novel membrane-based separation technology with the potential to produce pure water from high-salinity brine. It couples transport behaviors along the membrane and across the membrane. The brine in the feed is gradually concentrated due to the permeate flux across the membrane, which is a significant factor in initiating the scaling behavior on the membrane surface along the feed flow direction. It is of great interest to investigate and estimate the development of scaling on the membrane surface. This work specifically focuses on a long-distance membrane distillation process with a sodium chloride solution as the feed. A modeling approach has been developed to estimate the sodium chloride scaling development on the membrane surface along the flow direction. A set of experiments was conducted to validate the results. Based on mathematical simplification and analytical fitting, a simplified model was summarized to predict the initiating position of sodium chloride scaling on the membrane, which is meaningful for scaling control in industrial-scale applications of membrane distillation.

## 1. Introduction

Membrane distillation (MD) is a novel separation process with an important role in the field of treating high-salinity brine and zero liquid discharge (ZLD) processes [1,2,3]. Since the feed concentration has an insignificant influence on the permeate flux, MD is suitable for treating high-salinity brine [4,5]. The crystallization scaling process is easily initiated in the high-salinity brine treatment process. Crystallization scaling is one of the important problems in MD performance and industrial-scale applications [6,7]. The available crystallization scaling control methods in MD mainly include pretreatment, adding scale inhibitors, membrane surface bubbling, changing the membrane structure, and reverse MD processes [8,9,10,11]. Metal oxides are known for their anti-fouling and optical properties, which may improve MD performance. Incorporating metal oxides into membranes can delay the initiation of scaling, maintain a higher permeate flux, and enhance overall membrane durability [12,13,14].

In recent years, the combination of MD and crystallization technology has also been a promising brine treatment and ZLD technology [15,16,17,18]. There are spiral-wound MD modules and hollow-fiber MD modules for industrial-scale applications [19,20]. There are also industrial-scale applications for seawater treatment using MD modules [21,22]. The seawater treatment process operates continuously and stably without crystallization scaling inside the MD module [23].

The MD process usually has a long flow distance in industrial-scale applications [24,25]. Based on a previous study, the long-distance membrane distillation (LDMD) module is an effective way to explore the scaling process [26]. The temperature and concentration of the feed change simultaneously in the MD process. When the feed reaches the supersaturated condition, crystallization scaling is initiated [27,28,29,30]. The crystallization scaling process combines the transport along the tangential and normal direction of the membrane [31,32,33]. The membrane is the evaporation surface between two regions with different parameters. The probability of scaling also increases due to concentration polarization and temperature polarization [34,35]. Scaling on the membrane surface results in a decrease in the permeate flux, stoppage of the MD process, and even membrane wetting [36,37,38,39]. Quantitative prediction of the approximate initiating position of crystallization scaling is the key to operating the MD process continuously.

Sodium chloride (NaCl) is the most common and abundant salt in seawater and industrial wastewater [40]. Research on the MD process also focuses on the NaCl scaling process [41,42,43]. Based on the operating parameters and inherent characteristics of the membrane, the temperature distribution and concentration distribution in the NaCl scaling process are estimated by MATLAB iterations. A simplified prediction model is summarized to predict the initiating position of NaCl scaling in the LDMD module. Through a series of proof-of-principle tests, the simplified prediction model is validated. The gradual evolutionary process of NaCl scaling is also observed and characterized.

## 2. Experimental Section

### 2.1. Membrane Parameters

**Figure 1:**
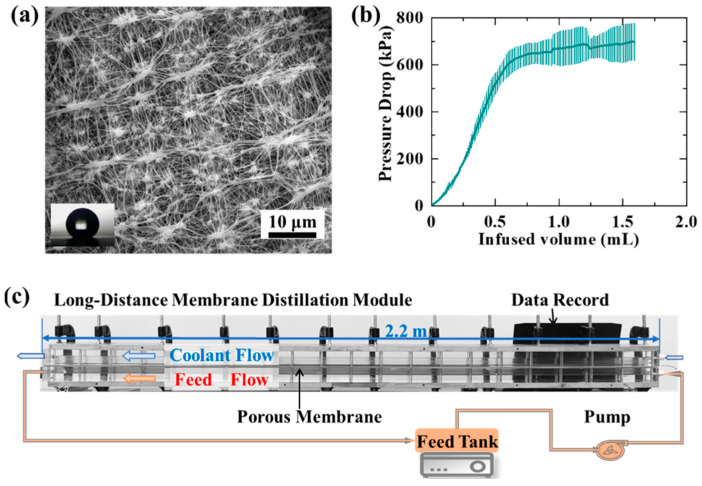
The micromorphology, LEP, and experimental set-up used in this work. (**a**) An SEM image of the PTFE membranes (nominal pore size: 0.22 μm, contact angle: 153 ± 5°). (**b**) The pressure drop of the test membranes. The LEP was tested by a syringe pump (0.5 mL/min). The value of the LEP is 610 ± 10 kPa. (**c**) The experimental set-up used in this work. The module length is 2040 mm. The module width is 40 mm. The module height is 25 mm. The width of the air gap is 2 mm.

PTFE membranes with an average pore diameter of 0.22 μm (Membrane Solutions, LLC, Auburn, WA, USA) were used in this work. A scanning electron microscope (TM 4000 Plus, HITACHI, Tokyo, Japan) was used to characterize the micromorphology of the membrane (see Figure 1a). The values of the liquid entry pressure (LEP) were tested with a self-made system from reference [44]. During the LEP tests, the syringe pump (0.5 mL/min) injected the deionized water into the membrane supporter. As shown in Figure 1b, the pressure data were recorded by the computer automatically. Membrane porosity was tested according to the following steps. During the tests, the overdried membranes were weighed and then immersed in isopropanol for 1 h. The membranes were taken out from isopropanol. The excess isopropanol on the membrane surface was wiped off. The membranes were weighed again. The ratio of the volume occupied by isopropanol to the membrane volume is the porosity [45]. The tests of membrane porosity were repeated 3 times. Deionized water was dropped (2.5 μL) on the membrane surface to test the contact angle with a goniometer (Yike-360A). A micrometer (211-101F) was used to measure the membrane thickness on 5 randomly selected membrane surfaces. The membrane parameters are shown in Table 1.

### 2.2. Long-Distance Membrane Distillation Module

As shown in Figure 1c, an air gap membrane distillation configuration is adopted in this work because of its high thermal efficiency. The width of the air gap is 2 mm. The module length is 2040 mm. The module width is 40 mm. The module height is 25 mm. The coolant channel is the same size as the feed channel. The sealing gasket is made of silica gel in the LDMD module. Twelve clamps were used to clamp and seal the LDMD module. The inclination angle was kept at 10 ± 1° to make the permeate flow out of the module in time. The membrane was loaded under the coolant in the LDMD module. The feed and coolant flow into each channel from bottom to top to make the module full of liquid. To reduce the probability of crystalline grain flowing into the feed channel, polyethylene mesh wrapped by polyacrylonitrile electrospun fibrous membranes was placed in the feed tank as a filter. The distance between the filaments of the polyethylene mesh is 1 mm. The angle between the filaments of the polyethylene mesh is 120°. The fiber diameter of the fibrous membranes is 1 μm.

### 2.3. Long-Distance Membrane Distillation Tests

The feed flows (1 ± 0.1 L/min) in the channel through a circulating pump (Guangquan, MP-15R). The initial temperature of the feed was set to 40 ± 1 °C, 50 ± 1 °C, and 55 ± 1 °C. The coolant temperature was kept at 20 ± 3 °C. The nearly saturated NaCl aqueous solution corresponding to the operating temperature was used as the feed. The volume of feed in the feed tank was kept at 3 ± 0.2 L. The permeate was weighed by an electronic balance. A chloride ion-selective electrode was used to test the permeate concentration.

### 2.4. Characterization of NaCl Scaling

The liquid was drained from the feed channel and the coolant channel after the tests. Dust-free papers were used to absorb the liquid drops remaining on the surface of the scaling and the membrane. The scaling dried in situ overnight naturally. The dried membranes were cut into ~14 cm to weigh the scaling. The mass of the scaling at the module inlet and outlet (~4 cm) was not weighed due to the unstable flow state.

## 3. Thermodynamic System

The concentration polarization and temperature polarization make it difficult to determine the concentration and temperature near the membrane surface. This work focuses on the approximate initiating position of the scaling on the membrane surface. To facilitate the estimation, the following assumptions are made:

(i) The concentration and temperature are considered to be uniform on the flow cross-section of the feed and the coolant.
(ii) When the feed reaches the supersaturated condition for heterogeneous nucleation, the scaling process is initiated on the membrane surface. The induction time of nucleation and the crystal growth process are not considered.
(iii) The heat transfer between the thermodynamic open system and the environment can be neglected. The MD process can be assumed as an adiabatic process when there is no heat input in the feed channel. The MD process can be assumed as an isothermal process after flow development in the entrance region due to the constant heat input along the feed channel.

**Figure 2:**
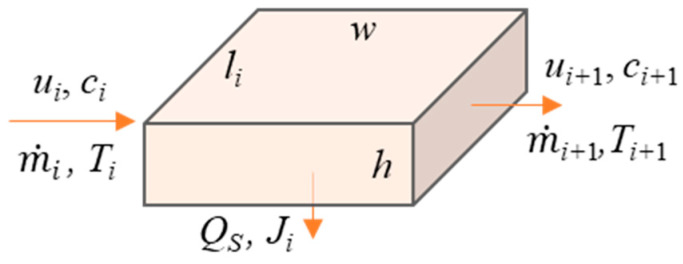
The specific region at the initiating position of NaCl scaling behavior in the feed is chosen as an open system in thermodynamics.

The feed parameters change constantly along the flow direction in the LDMD module due to evaporation and heat transfer. As shown in Figure 2, the distance that the feed flows per second is taken as the length of the thermodynamic open system to facilitate the estimation. According to the mass conservation,

(1) $${\dot{m}}_{i}-{\dot{m}}_{i+1}=J_{i}wl_{i}$$

where ${\dot{m}}_{i}$ and ${\dot{m}}_{i+1}$ are the mass flow rates of the inlet and the outlet in the thermodynamic open system, respectively. *J_i_* is the permeate flux of the thermodynamic open system. *l_i_* and *w* are the length and the width of the thermodynamic open system, respectively. *i* is the number of each computational unit used in the estimation program, which is artificially defined as the number of thermodynamic open systems. According to the solute mass conservation, the concentration at the feed outlet is described by Equation (2):

(2) $$c_{i+1}=\frac{{{\dot{m}}_{i}c}_{i}}{{\dot{m}}_{i+1}}$$

where ${\dot{m}}_{i}$ and ${\dot{m}}_{i+1}$ are the mass flow rates of the inlet and the outlet in the thermodynamic open system, respectively. *c_i_* and *c_i_*_+1_ are the concentrations at the inlet and the outlet in the thermodynamic open system, respectively. According to the energy conservation of the thermodynamic open system, the outlet temperature is calculated by Equation (3):

(3) $$T_{i+1}=T_{i}-\frac{J_{i}\Delta H}{\rho_{i}hc_{p}\eta_{i}}$$

where *T_i_* and *T_i_*_+1_ are the temperatures at the inlet and the outlet in the thermodynamic open system, respectively. *J_i_* is the permeate flux of the thermodynamic open system. Δ*H* is the latent heat of vaporization. *c_p_* is the specific heat capacity. *η_i_* is the thermal efficiency of the thermodynamic open system.

## 4. Results and Discussion

### 4.1. Theoretical Estimation of NaCl Scaling Process

**Figure 3:**
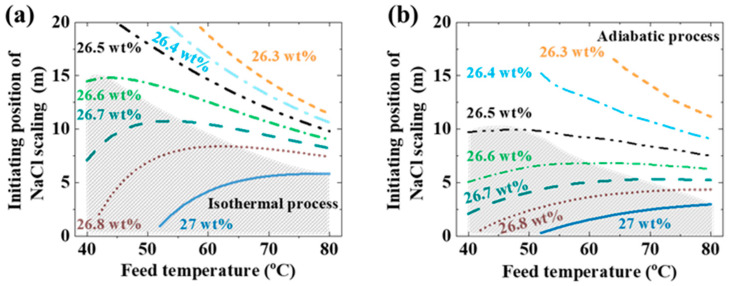
The theoretical estimated initiating position of the scaling with a NaCl aqueous solution under (**a**) the isothermal process and (**b**) the adiabatic process. The theoretical estimation was based on the following parameters: feed flow rate: 1 L/min, air gap width: 2 mm, *B* = 5 × 10^−8^ s/m, *w* = 40 mm, and *h* = 25 mm.

Figure 3a,b show the initiating position of the scaling in the LDMD module under the isothermal process and the adiabatic process, respectively. The estimation is based on the membrane parameters and the operating parameters in Section 2. The initiating position of the scaling is proportional to the initial concentration of the feed. The feed concentration has an insignificant effect on the permeate flux under the estimated conditions. The initial concentration of the feed becomes the only factor determining the initiating position of the scaling under the same initial temperature of the feed. The local supersaturated state of the feed is also determined by the local temperature. In the unshaded areas in Figure 3, the high initial temperature of the feed shortens the initiating position of the scaling under the same initial concentration of the feed. The feed concentration is much lower than the supersaturated concentration corresponding to the initial temperature of the feed. More solvent needs to be evaporated under these conditions. The permeate flux is the dominant factor. The effect of the supersaturated concentration caused by the decreasing feed temperature is relatively small. In the shaded area in Figure 3, the initial concentration of the feed is nearly saturated. The supersaturated concentration is the dominant factor.

The scaling process will happen in the feed at a certain distance in the LDMD module with an infinite length in the isothermal process. When the initial concentration of the feed is less than 26 wt%, the scaling process will not happen in the adiabatic process with a channel height higher than 25 mm and a feed temperature less than 80 °C. The feed temperature decreases continuously until the feed temperature is close to the coolant temperature. Thus, the MD process stops in the adiabatic process under the same LDMD conditions. The outlet feed concentration may be less than the supersaturated concentration. The initiating position of the scaling is shorter in the isothermal process with the initial concentration of the feed less than 26.3 wt%. More solvent needs to be evaporated at a lower feed concentration. The feed temperature leads to a higher permeate flux in the isothermal process than that in the adiabatic process. The initiating position of the scaling in the adiabatic process is shorter with the initial concentration of the feed equal to or higher than 26.3 wt%. The supersaturated state is the dominant factor in the scaling process under these conditions.

### 4.2. Simplified Estimation of Initiating Position of NaCl Scaling

(i) The isothermal process

According to Equation (1), when the feed reaches the supersaturated condition, the initiating position of scaling can be estimated from the following:

(4) $$L=\sum_{i=1}^{n}\frac{{\dot{m}}_{i}-{\dot{m}}_{i+1}}{J_{i}w}$$

where ${\dot{m}}_{i}$ and ${\dot{m}}_{i+1}$ are the mass flow rates of the inlet and the outlet in the thermodynamic open system, respectively. *J_i_* is the permeate flux of the thermodynamic open system. *w* is the width of the thermodynamic open system. The feed temperature and the supersaturated concentration remain unchanged in the isothermal process. The feed concentration has an insignificant influence on the saturated water vapor pressure. Therefore, the permeate flux can be considered to have the same value. Combining Equation (2), Equation (4), and the simplified equation of the permeate flux from [46], the initiating position of scaling can be estimated from the following:

(5) $$L=\frac{\rho hu}{1000BEXP(T_{0}/20)}\left(1-\frac{c_{0}}{c^{*}}\right)$$

where *T*_0_ is the initial temperature of the feed. *h* is the module height. *u* is the flow rate at the module inlet. *ρ* is the feed density. c* is the supersaturated concentration corresponding to the local temperature. *c*_0_ is the initial feed concentration. *B* is the mass transfer coefficient.

(ii) The adiabatic process

**Figure 4:**
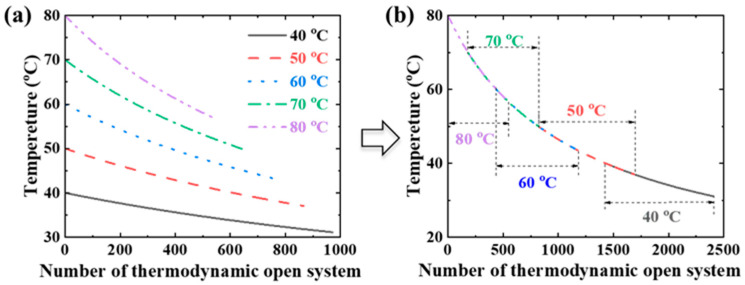
The temperature distribution in the feed channel under the same estimated conditions. (**a**) The temperature distribution with the number of thermodynamic open systems. (**b**) The temperature distribution after translating the curves. The following parameters were used for the theoretical estimation: feed flow rate: 1 L/min, air gap width: 2 mm, *B* = 5 × 10^−8^ s/m, *w* = 40 mm, and *h* = 25 mm.

The scaling process does not happen in the adiabatic process with the initial concentration of the feed lower than 26 wt%, according to the theoretical estimation. The permeate flux in the MD process is generally less than 100 kg/m^2^/h. The mass of the permeate is less than 0.1% of the mass in the thermodynamic open system with the flow channel higher than 1 mm. Therefore, the feed parameters Δ*H*, *u*, *c_p_*, and *ρ* can be considered constant values in the adiabatic process. The inlet feed temperature instead of the average temperature was used to estimate the permeate flux and thermal efficiency in this study. According to Equation (3), the outlet temperature is only a function of the initial temperature in the thermodynamic open system with the same module height. Therefore, the MD processes with different initial temperatures of the feed flowing through the same height channel can be regarded as different parts of the same MD process. As shown in Figure 4, all of the curves coincide with different segments of one curve after translating the curves. Based on the above simplification, the relationships of the total permeate flux and the initiating position of scaling with feed temperature are obtained from the estimation and curve fitting:

(6) $$L=1.1\frac{uh}{B}\left(T_{out}^{-1.5}-T_{0}^{-1.5}\right)$$

(7) $$T_{out}={\left(T_{0}^{1.5}-\frac{{\Delta J}_{t}}{0.1h}\right)}^{1/1.5}$$

where Δ*J_t_* is the total permeate flux. *T_out_* is the outlet temperature of the feed. *L* is the initiating position of the scaling. *T*_0_ is the initial temperature of the feed. *h* is the module height. *u* is the feed flow rate. *B* is the mass transfer coefficient. When the feed reaches a supersaturated state, the total permeate flux can be estimated from Equation (8):

(8) $${\Delta J}_{t}=\rho h\left(1-\frac{c_{0}}{c^{*}}\right)$$

where Δ*J_t_* is the total permeate flux. *ρ* is the feed density. *h* is the module height. c* is the supersaturated concentration. *c*_0_ is the initial feed concentration.

The channel width and feed flow rate have an insignificant influence on c* according to the theoretical estimation. The change in c* is less than 0.2% when the channel width is in the range of 10–50 mm and the *B* value is in the range of 3 × 10^−8^–3 × 10^−7^ s/m. c* is mainly affected by *T*_0_ and *c*_0_. c* in the adiabatic process can be estimated from Equation (9):

(9) $$c^{*}=1\times 10^{-4}T_{0}+0.6c_{0}+0.105$$

where *T*_0_ is the initial temperature of the feed. *c*_0_ is the initial concentration of the feed.

The estimation is divided into four steps.

(i) Estimate the supersaturated concentration corresponding to the local temperature (c*) from Equation (9).
(ii) Estimate the total permeate flux (Δ*J_t_*) from Equation (8).
(iii) Estimate the outlet feed temperature of the LDMD module (*T_out_*) from Equation (7).
(iv) Estimate the initiating position of the scaling (*L*) from Equation (6).

If the outlet feed temperature (*T_out_*) is less than or equal to the coolant temperature, it is considered that the scaling process does not happen inside the LDMD module. The simplified estimation flow charts of the isothermal process and the adiabatic process are shown in Table 2.

### 4.3. Evolutionary Process of NaCl Scaling

**Figure 5:**
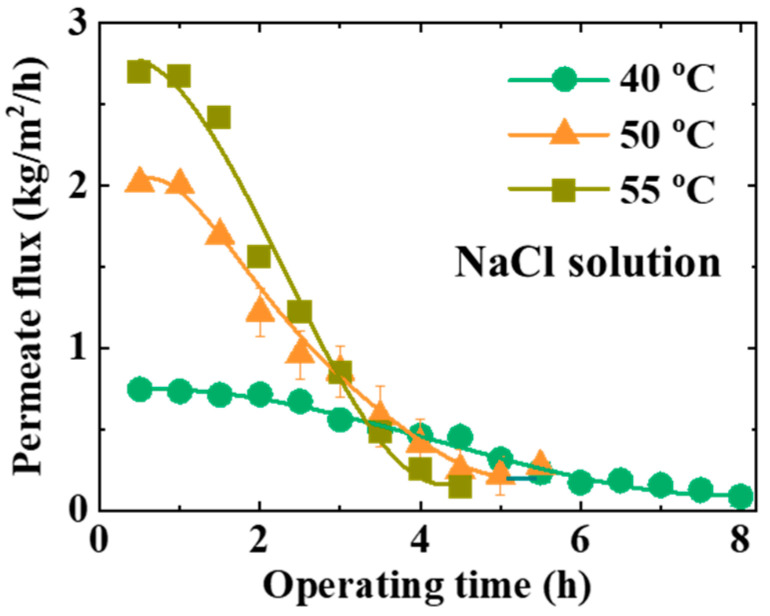
The LDMD performance with a NaCl solution at different parameters. The feed flow rate was kept at 1 ± 0.1 L/min. The coolant temperature was set to 20 ± 3 °C.

As shown in Figure 5, the permeate flux with the NaCl aqueous solution decreases over time until the scaling covers the membrane surface. The values of the initiating position of the scaling are ~90 m, ~60 m, and ~60 m with the initial temperature of the feed at 40 °C, 50 °C, and 55 °C, respectively. The values of the initiating position of the scaling estimated from the simplified module are 41 m, 26 m, and 21 m, respectively. The trend of the theoretical estimation is consistent with that of the experimental data. The initiating position of the scaling of the experimental data is longer than that of the theoretical estimation. The estimated initiating position of the scaling is the distance required for the feed to reach the supersaturated state. According to the diffusion-controlled process, the induction time of crystals with a visible size of 0.1 mm is about 1000 s [47]. The corresponding flow distance in this work is ~16 m. The crystal nucleus may attach to the membrane and module wall, flow with the feed solution, and be filtered in the feed tank. Therefore, a longer operating time is needed to observe the scaling on the membrane surface in the experiments.

There was no scaling on the membrane surface at the beginning of the LDMD tests. The values of the permeate flux are 0.5, 2, and 2.7 kg/m^2^/h with a feed temperature of 40 °C, 50 °C, and 55 °C, respectively. The values of the mass transfer coefficient are 4.1 × 10^−8^ s/m, 5.2 × 10^−8^ s/m, and 5.2 × 10^−8^ s/m, respectively. The gradual evolutionary process of the scaling is similar to that of copper sulfate scaling in the LDMD module, as discussed in our previous study [26]. The feed concentration increases continuously toward the supersaturated state until the scaling process is initiated. The scaling on the membrane surface grows continuously. The porous membrane is gradually covered by the scaling, leading to pore clogging of the vapor diffusion channel. Thus, the effective evaporation area of the membrane is gradually reduced, leading to a gradual decline in the permeate flux over time. A higher initial temperature of the feed shortens the flow distance to the initiating position of the scaling, resulting in a shorter time to stop the permeate flux. The solubility of the sodium chloride solution can be neglected with temperature because of the nearly saturated state of the solution in all tests. The supersaturation state is mainly related to solvent loss caused by evaporation. The feed solution with a higher temperature gives a larger transmembrane flux initially because of the larger driving force from the saturated pressure difference across the porous membrane. Thus, higher feed temperatures also lead to a shorter flow distance to the initiating position of the scaling.

### 4.4. Behaviors of NaCl Scaling

**Figure 6:**
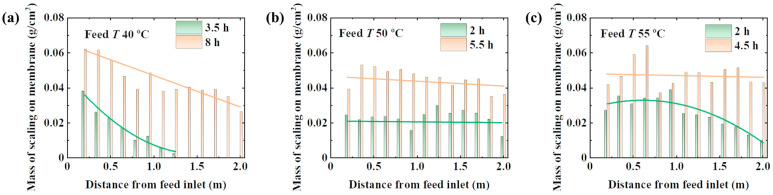
The behaviors of NaCl scaling in the LDMD module at different operating times. The feed temperature was set to (**a**) 40 ± 1 °C, (**b**) 50 ± 1 °C, and (**c**) 55 ± 1 °C. The feed flow rate was kept at 1 ± 0.1 L/min. The coolant temperature was set to 20 ± 3 °C.

As shown in Figure 6, the mass of the scaling is proportional to the operating time of the MD process. To characterize the scaling process, the operating time when the permeate flux drops to a half value or zero is selected. The mass of the scaling at a feed temperature of 40 °C decreases along the flow direction, as shown in Figure 6a. This indicates that the scaling is easy to accumulate near the entrance of the module. The largest temperature gradient across the membrane near the entrance leads to the highest permeate flux. Thus, the feed concentration increases significantly. The exposed membrane surface gradually scales and conducts to the module outlet in turn. Due to the heat dissipation and the latent heat transferred with the permeate flux, the feed temperature decreases. According to the Antoine equation, the saturated vapor pressure is exponential with the temperature. Thus, the supersaturated state of the feed along the flow direction decreases exponentially. This is consistent with the experimental data under 4 h of operation, as shown in Figure 6a. With the increasing operating time, the membrane surface is gradually covered with the scaling, leading to the stoppage of LDMD. The supersaturated state near the membrane surface completely covered with the scaling is only determined by the temperature reduction caused by heat dissipation. Therefore, the growth rate of the scaling on the scaling-covered membrane surface decreases. The mass of the scaling on the completely covered membrane surface tends to be uniform after a long operating time.

As shown in Figure 6, a short time is required for the scaling to cover the membrane surface completely at a high feed temperature. This indicates that the growth rate the of scaling is high at a high feed temperature. The mass of the scaling tends to be uniform (0.05 ± 0.01 g/cm^2^) at all test temperatures when the membrane surface is completely covered. This may be due to the little variation in the saturated concentration of NaCl (27 wt%–27.5 wt%) and the small relative supersaturation for homogeneous crystallization (1.2 × 10^−4^) [47]. A higher feed temperature may lead to heterogeneous or even homogeneous nucleation on all membrane surfaces at the same time. Therefore, the mass of the scaling on the membrane surface is uniform under 2 h of operation with feed temperatures of 50 °C and 55 °C. A high initial temperature of the feed reduces the size of the scaling due to a high supersaturated state of the feed. It is easy for the small particles of the scaling to be washed away from the membrane surface by the unstable fluid at the module entrance. Therefore, the mass of the scaling at the module entrance decreases at a feed temperature of 55 °C under 4.5 h of operation.

## 5. Conclusions

This paper focuses on the gradual evolutionary process of NaCl scaling and the initial position of NaCl scaling. The estimation of the initiating position of NaCl scaling in the MD process with a NaCl aqueous solution was established. A practical model based on mathematical simplification and analytical fitting was proposed. Based on the operating parameters and inherent membrane characteristics, the temperature and concentration along the flow direction were estimated by MATLAB iterations. The temperature distribution in the feed channel is independent of the module width. The verification tests show that the trend of the initiating position of NaCl scaling is the same as that of the estimated value. The high permeate flux leads to a short initiating position of NaCl scaling on the membrane surface. The mass of NaCl scaling on the completely covered membrane surface tends to be uniform. The simplified estimation model is verified to effectively predict the flow distance without NaCl scaling. The NaCl scaling process will not happen in the adiabatic process in a common LDMD module with an initial concentration less than 26 wt% and an initial temperature less than 80 °C. The initiating position of NaCl scaling in the adiabatic process can be estimated by one simplified equation. This provides a potential tool for LDMD module design and failure analysis in the treatment of NaCl aqueous solutions.

## Figures and Tables

**Table 1:** Membrane parameters.

Membrane Thickness	Contact Angle	LEP	Porosity
60 ± 5 μm	153 ± 5°	600 ± 50 kPa	75 ± 5%

**Table 2:** Estimation flow charts of the isothermal process and the adiabatic process.

Isothermal Process	Adiabatic Process
Equation (5)	Equation (9) Equation (8) Equation (7) Equation (6)

## Data Availability

The original contributions presented in the study are included in the article, further inquiries can be directed to the corresponding author.
